# Polymorphisms in *GSTT1*, *GSTZ1*, and *CYP2E1*, Disinfection By-products, and Risk of Bladder Cancer in Spain

**DOI:** 10.1289/ehp.1002206

**Published:** 2010-09-12

**Authors:** Kenneth P. Cantor, Cristina M. Villanueva, Debra T. Silverman, Jonine D. Figueroa, Francisco X. Real, Monserrat Garcia-Closas, Nuria Malats, Stephen Chanock, Meredith Yeager, Adonina Tardon, Reina Garcia-Closas, Consol Serra, Alfredo Carrato, Gemma Castaño-Vinyals, Claudine Samanic, Nathaniel Rothman, Manolis Kogevinas

**Affiliations:** 1 Division of Cancer Epidemiology and Genetics, National Cancer Institute, Bethesda, Maryland, USA; 2 KP Cantor Environmental LLC, Silver Spring, Maryland, USA; 3 Centre for Research in Environmental Epidemiology, Barcelona, Spain; 4 Institut Municipal d’Investigacio Medica–Hospital del Mar, Barcelona, Spain; 5 CIBER Epidemiologia y Salud Publica, Barcelona, Spain; 6 Centro Nacional de Investigaciones Oncológicas, Madrid, Spain; 7 Universidad Pompeu Fabra, Barcelona, Spain; 8 Universidad de Oviedo, Oviedo, Spain; 9 Unidad de Invesigación, Hospital Universitario de Canarias, La Laguna, Spain; 10 Consorci Hospitalari Parc Tauli, Sabadell, Spain; 11 Ramon y Cajal University Hospital, Madrid, Spain; 12 National School of Public Health, Athens, Greece

**Keywords:** bladder cancer, CYP2E1, disinfection by-products, drinking water, GSTT1, GSTZ1, trihalomethanes

## Abstract

**Background:**

Bladder cancer has been linked with long-term exposure to disinfection by-products (DBPs) in drinking water.

**Objectives:**

In this study we investigated the combined influence of DBP exposure and polymorphisms in glutathione *S*-transferase (*GSTT1*, *GSTZ1*) and cytochrome P450 (*CYP2E1*) genes in the metabolic pathways of selected by-products on bladder cancer in a hospital-based case–control study in Spain.

**Methods:**

Average exposures to trihalomethanes (THMs; a surrogate for DBPs) from 15 years of age were estimated for each subject based on residential history and information on municipal water sources among 680 cases and 714 controls. We estimated effects of THMs and *GSTT1*, *GSTZ1*, and *CYP2E1* polymorphisms on bladder cancer using adjusted logistic regression models with and without interaction terms.

**Results:**

THM exposure was positively associated with bladder cancer: adjusted odds ratios (ORs) and 95% confidence intervals (CIs) were 1.2 (0.8–1.8), 1.8 (1.1–2.9), and 1.8 (0.9–3.5) for THM quartiles 2, 3, and 4, respectively, relative to quartile 1. Associations between THMs and bladder cancer were stronger among subjects who were *GSTT1* +/+ or +/− versus *GSTT1* null (*p*_interaction_ = 0.021), *GSTZ1* rs1046428 CT/TT versus CC (*p*_interaction_ = 0.018), or *CYP2E1* rs2031920 CC versus CT/TT (*p*_interaction_ = 0.035). Among the 195 cases and 192 controls with high-risk forms of *GSTT1* and *GSTZ1,* the ORs for quartiles 2, 3, and 4 of THMs were 1.5 (0.7–3.5), 3.4 (1.4–8.2), and 5.9 (1.8–19.0), respectively.

**Conclusions:**

Polymorphisms in key metabolizing enzymes modified DBP-associated bladder cancer risk. The consistency of these findings with experimental observations of GSTT1, GSTZ1, and CYP2E1 activity strengthens the hypothesis that DBPs cause bladder cancer and suggests possible mechanisms as well as the classes of compounds likely to be implicated.

Chlorine is a cost-effective drinking water disinfectant that has been used since the early twentieth century to control a panoply of waterborne infectious diseases. By-products of the interaction of chlorine with organic precursors in water were first noted in 1974, with the discovery of trihalomethanes (THMs) in disinfected water (Bellar and Lichtenberg 1974; Rook 1974). Since then, hundreds of halogenated chemical species in the disinfection by-product (DBP) mixture have been detected, including both brominated and chlorinated compounds (Richardson 2003). THMs and haloacetic acids (HAAs) are the chemical groups at highest concentration in most by-product mixtures. Toxicological and epidemiologic studies of DBPs provide evidence of elevated risk of cancer and adverse birth outcomes (Cantor et al. 2006; Grellier et al. 2010; Nieuwenhuijsen et al. 2009). In particular, human bladder cancer has been consistently linked with long-term exposure (Cantor et al. 1998; King and Marrett 1996; McGeehin et al. 1993; Villanueva et al. 2004). These observations are supported by evidence of mutagenicity of the mixture and carcinogenicity of some constituents (Komulainen 2004; Richardson et al. 2007). To date, the role of genetic variability in modulating adverse health effects of DBPs has received limited attention (Infante-Rivard et al. 2002), and bladder cancer has not been studied in this regard.

At least three enzymes in the metabolic pathways of DBP components are candidates for examination. Glutathione *S*-transferase (GST) theta-1 (GSTT1) activates brominated THMs to mutagens in a transgenic strain of *Salmonella* (DeMarini et al. 1997; Pegram et al. 1997). GST zeta-1 (GSTZ1) catalyzes the oxygenation of dichloro- and other α-haloacids, some of which are animal carcinogens (DeAngelo et al. 1999; Melnick et al. 2007; Tong et al. 1998). Cytochrome P450 2E1 (CYP2E1) metabolizes a wide variety of aliphatic hydrocarbons, solvents, and industrial monomers (Guengerich et al. 1991) and is responsible for the primary oxidation of THMs. Genes that code for these enzymes are polymorphic in human populations, with the *GSTT1* deletion resulting in lack of enzyme activity, and with several nonsynonymous single-nucleotide polymorphisms (SNPs) in *GSTZ1* and *CYP2E1* resulting in modified enzymatic activity (Blackburn et al. 2001; Bolt et al. 2003). We hypothesized that one or more of these functional polymorphisms could influence bladder cancer risk posed by DBPs, and we investigated this in a large case–control study in Spain, where in a previous study (Villanueva et al. 2007) we observed elevated risk of bladder cancer after long-term exposure to DBPs.

## Materials and Methods

We conducted a hospital-based case–control study in 18 hospitals located in five areas of Spain [Asturias, Barcelona metropolitan area, Valles/Bages (including the municipalities of Manresa and Sabadell), Alicante, and Tenerife] (Appendix 1). Eligible cases were 21–80 years of age, newly diagnosed with histologically confirmed urothelial carcinoma of the bladder between 1998 and 2001, and living in the catchment geographic area of participating hospitals. Cases were identified from the registers of urological services augmented by regular and frequent evaluations of hospital discharge records, pathology records, and local cancer registries. A panel of expert pathologists confirmed diagnoses and ensured uniformity of classification criteria, based on the 1998 World Health Organization/International Society of Urological Pathology system (Epstein et al. 1998).

Controls were selected from patients admitted to participating hospitals with conditions thought to be unrelated to the major risk factors of bladder cancer, such as tobacco use. Diagnostic categories for controls were as follows: 37% hernias, 11% other abdominal surgery, 23% fractures, 7% other orthopedic problems, 12% hydrocoele, 4% circulatory disorders, 2% dermatological disorders, 1% ophthalmological disorders, and 3% other diseases. Controls were individually matched to cases by age at interview within 5-year strata, sex, ethnic origin, and hospital catchment area, a well-defined area corresponding to the specific health services region covered by each hospital. Written informed consent was obtained from each subject before the study. The study was approved by the review board of each participating institution and in accord with an assurance filed with and approved by the U.S. Department of Health and Human Services.

### Individual data

After obtaining informed consent, trained interviewers administered a computer-assisted personal interview (CAPI) to participants during their hospital stay. Interview items included sociodemographic characteristics; smoking habits; occupational, residential, and medical histories; and familial history of cancer. We identified 1,457 eligible cases and 1,465 eligible controls. Of these, 84% of cases (*n* = 1,219) and 87% of controls (*n* = 1,271) participated. Subjects who refused to answer the CAPI were administered a reduced interview of critical items (21% of cases and 19% of controls). Questionnaire information on water-related exposures used in this analysis included residential history from birth [all residences of at least 1 year, drinking water source at each residence (municipal/bottled/private well/other)] and swimming pool use as an adult. These data were collected from all participants, including those who responded to the critical items questionnaire.

### Exposure data

Using a structured questionnaire, we collected historic water quality data from approximately 200 local authorities and 150 water companies in the study areas. For 123 study municipalities, covering 78.5% of the total study exposure-years, we obtained annual average THM levels. In addition, one of us (C.M.V.) measured levels of the four THMs [chloroform, bromodichloromethane (BDCM), dibromochloromethane, and bromoform] in 113 tap water samples from the studied geographic areas between September and December 1999.

Average THM levels in recent years were extrapolated back to approximately 1920. Historical THM levels were estimated by municipality under the assumption that past THM levels were similar to current concentrations when the water source had not changed. When the water source had changed, we calculated the average THM level using the proportion of surface water during the relevant time period. We assumed that the THM level before the start of chlorination was zero. The exposure assessment was described previously (Villanueva et al. 2006, 2007).

### Lifetime individual exposure indices

We merged individual and municipal databases with individual residential information by year and municipality, and obtained individual year-by-year average THM levels, water source, and chlorination status for each study subject. We used as an exposure metric the average THM level of the water source serving participant residences in the period between age 15 years and the interview, as described previously in the analysis of the main effects of DBP exposure (Villanueva et al. 2007). As in that study, we restricted our analysis to subjects with a household THM estimate for at least 70% of the years in this exposure window.

### Genotyping

DNA for genotype assays was extracted from leukocytes or mouthwash samples as described previously (Garcia-Closas et al. 2005). Genotype assays for polymorphisms in *GSTT1*, *GSTM1*, and *N*-acetyltransferase 2 (*NAT2*) were conducted at the Core Genotyping Facility of the Division of Cancer Epidemiology and Genetics (National Cancer Institute). The SNPs of *NAT2* and assignments of rapid/intermediate/slow acetylator types, as well as the *GSTM1* genotypes, have been described in detail elsewhere (Garcia-Closas et al. 2005). *GSTT1* genotypes were defined as null (−/−) if a deletion was found in both copies of the gene and present if one (+/−) or none (+/+) of the copies had a deletion. We used the TaqMan assay (Applied Biosystems, Foster City, CA, USA) for SNPs in *NAT2*, two of three SNPs in *GSTZ1* [E32K (rs7975) and G42R (rs7972)], and deletions in *GSTT1* and *GSTM1*. The methods for each specific assay are available from the National Cancer Institute (2010). A third SNP in *GSTZ1* (T82M rs1046428) and three SNPs in *CYP2E1* (IVS7–118C>G rs2070676, −1054C>T rs2031920, and −1514G>T rs8192766) were determined in a GoldenGate assay (Illumina, San Diego, CA, USA) (Garcia-Closas et al. 2007). All genotypes studied were in Hardy-Weinberg equilibrium in the control population. Duplicate quality control samples (*n* = 93 pairs) showed ≥ 99% agreement for all assays.

### Statistical analysis

We considered results to be statistically significant if the alpha level was ≤ 0.05. Subjects were grouped by quartile of the time-weighted average THM level since age 15 years. We used unconditional logistic regression to calculate odds ratios (ORs) and 95% confidence intervals (CIs). ORs were adjusted for age (continuous), sex, smoking status (never/former/current), size of the municipality of longest residence until 18 years of age (reported by the participant as metropolis/city, small city, village, or farm), education (three strata: less than primary school, less than high school, and high school or more), geographic area (six strata: Barcelona, Sabadell, Manresa, Alicante, Tenerife, and Asturias), and overall quality of interview (reported by interviewer as unsatisfactory, questionable, reliable, or high quality). Analyses using more detailed smoking information (total duration, average daily number of cigarettes, tobacco type, pack-years) gave similar results and are not reported. Subjects were defined as “former” smokers if they had quit at least 1 year before the interview. Missing data for covariates were coded to a separate category for each variable included in the models. This applied to a small proportion of respondents for each covariate. For example, < 1% of respondents were missing data on smoking status (Samanic et al. 2006). Linear *p*-trends were calculated using a likelihood ratio test comparing the model with and without the THM exposure variable modeled as a continuous variable with each quartile coded according to its median value.

Interactions between genotypes and exposure to DBPs, as estimated by average THM levels serving the household between 15 years of age and diagnosis (cases) or interview (controls), were also tested using the likelihood-ratio test to allow estimation of parameters under the assumption of genotype–DBP (THMs) independence in the source population. Tests for multiplicative interaction were used to assess whether the genotype ORs within categories of DBP exposure or, equivalently, DBP ORs within genotype categories differed significantly from each other. Haplotype frequencies for *GSTZ1* and *CYP2E1* were estimated using HaploStats (version 1.2.1; http://mayoresearch.mayo.edu/mayo/research/biostat/schaid.cfm) using the program language R (http://www.r-project.org/).

## Results

Of the 1,219 cases and 1,271 controls interviewed, 1,188 (97%) cases and 1,173 (92%) controls provided a blood or buccal cell sample for DNA extraction. After excluding cases and controls with low amounts of DNA, nonwhite individuals (to limit heterogeneity), and DNA quality control difficulties, the study population with adequate genetic material available for analysis numbered 1,150 cases and 1,149 controls (Garcia-Closas et al. 2005). After further excluding individuals with inadequate exposure data as described above, the maximum numbers with genotyping data were 680 cases (595 males, 85 females) and 714 controls (622 males, 92 females). In each gene-specific analysis, we applied additional exclusions because of incomplete genetic information.

The ORs (95% CIs) for former [2.7 (1.9–3.7)] and current [5.9 (4.2–8.5)] smokers of cigarettes in the analysis data set, relative to never-smokers, paralleled those found in the full study population (Samanic et al. 2006). The ORs (95% CIs) by increasing quartile of THMs were 1.0 (referent), 1.2 (0.8–1.9), 1.8 (1.1–2.9), and 1.8 (0.9–3.5) (*p*_trend_ = 0.029) (Table 1), similar to the findings of Villanueva et al. (2007). These ORs are adjusted for the covariates listed above, as are all subsequent ORs reported here. Findings from Garcia-Closas et al. (2005), from this study population, showed significant associations between bladder cancer and *NAT2* slow acetylator compared with rapid/intermediate genotypes [OR (95% CI) of 1.4 (1.2–1.7)] and *GSTM1* null (−/−) versus *GSTM1* present genotypes [1.7 (1.4–2.0)]. Estimates in the present analyses were similar, with *NAT2* (slow compared with rapid plus intermediate acetylator type) having an OR (95% CI) of 1.33 (1.06–1.68), and with *GSTM1* null compared with the *GSTM1* present genotype having an OR (95% CI) of 1.77 (1.41–2.22). We found slightly elevated, nonsignificant risks for *GSTT1* presence compared with null [1.21 (0.92–1.59)]. The association for the inverse relationship (null vs. presence) was 0.81 (0.61–1.09). In the full study, compared with the homozygous presence of *GSTT1*, the ORs (95% CIs) were 1.2 (1.0–1.5) for heterozygotes (+/−) and 1.0 (0.8–1.3) for homozygous null (−/−) (Garcia-Closas et al. 2005).

We found nonsignificant elevations or decreases in risk for the three functional SNPs of *GSTZ1* and the three variants of *CYP2E1* that we tested. Associations for the three SNPs in *GSTZ1* and the three in *CYP2E1* have not been reported previously. The *GSTZ1* SNPs were in linkage disequilibrium (pairwise *D*′ values ranged from 0.98 to 1.0); however, they were not highly correlated with each other (*r*^2^ ranged from 0.02 to 0.18). Of the three *CYP2E1* SNPs tested, rs8192766 was not in linkage disequilibrium with rs2070676 (*D*′ = 0.34). The other two pairs were in linkage disequilibrium (*D*′ ≥ 0.98). None of the three *CYP2E1* SNPs was highly correlated with any other (*r*^2^ ranged from 0.006 to 0.34). Haplotype analysis for *GSTZ1* and the three SNPs genotyped revealed no common haplotype significantly associated with risk (global *p* = 0.80; data not shown). We made similar observations for *CYP2E1* (global *p* = 0.76).

We observed significant interactions with THMs for *GSTT1* (present vs. null) (*p*_interaction_ = 0.021), exon 7 +29T>C rs1046428 in *GSTZ1* (*p*_interaction_ = 0.018), and −1054C>T rs2031920 in *CYP2E1* (*p*_interaction_ = 0.035; Table 2) but not for the other two SNPs in *GSTZ1* that we tested (rs7975, rs7972) or for the two other SNPs tested in *CYP2E1* (rs2070676, rs8192766; data not shown). We found no significant trends in associations with increasing THM quartiles among subjects with the *GSTT1* null, *GSTZ1* CC, or *CYP2E1* CT/TT genotypes; however, we did find significant positive trends with increasing THM exposure among subjects with the *GSTT1* present genotype *GSTZ1* CT/TT, or *CYP2E1* CC (Table 2). Haplotype analysis for *GSTZ1* and *CYP2E1* did not provide additional information beyond the single SNP analysis (data not shown). We found no evidence of interaction between either *NAT2* slow acetylator or *GSTM1*-null genotype and long-term average THM level (*p*_interaction_ = 0.95 and 0.79, respectively; data not shown).

Because of small numbers of subjects (37 cases, 42 controls) with the CT variant of *CYP2E1* rs2031920 (no subjects carried the TT variant), it was not possible to analyze a three-way interaction that included these subjects. We found a significant interaction between average THM level and combined *GSTT1* and *GSTZ1* rs1046428 genotypes (*p*_interaction_ = 0.0052) in analyses that included subjects with both the variant and common forms of *CYP2E1* rs2031920 (Table 3). Among subjects with both *GSTT1* present and *GSTZ1* CT/TT genotypes, OR increased monotonically to 5.9 (95% CI, 1.8–19.0) in the highest quartile of THMs (*p*_trend_ = 0.0012). In contrast, among subjects with both *GSTT1* null and *GSTZ1* CC genotypes, we found no increase in relative risk with increasing THM level (*p*_interaction_ = 0.0052). These analyses excluded subjects with both low-risk variants of *GSTZ1* and high-risk variants of *GSTT1*, or vice versa. When we restricted this analysis to subjects with the common form (CC) of *CYP2E1* rs2031920, among those with both *GSTT1* present and *GSTZ1* CT/TT genotypes, the OR increased monotonically to 9.3 (2.5–34.0) in the highest quartile of THM exposure (> 49.0 μg/L), relative to participants with these genotypes in the lowest quartile [≤ 8.0 μg/L; *p*_trend_ = 0.0006 with *p*_interaction_ = 0.010; see Supplemental Material, Table 1 (doi:10.1289/ehp.1002206)].

We calculated the main effects for the respective SNPs within increasing quartile strata of long-term average THMs (Table 4) where we found increasing relative risks for bladder cancer for *GSTT1* present, *GSTZ1* rs1046428 *CT/TT*, and *CYP2E1* rs2031920 CC. Among subjects with exposure to THMs in the highest quartile, the ORs were elevated and the 95% CIs excluded 1.0 for *GSTT1* present (vs. *GSTT1* null), *GSTZ1* (CT/TT vs. CC), and *CYP2E1* (CC vs. CT/TT), whereas ORs for these polymorphisms were close to 1.0 among subjects in the lowest THM exposure quartile. The associations with bladder cancer risk for *NAT2* slow versus rapid/intermediate acetylator and *GSTM1* null versus present within each THM stratum were variable and consistent with the overall elevated main effects for these genes.

In earlier analyses of these data, we found a significant association with ever swimming in pools [OR = 1.62 (95% CI, 1.20–2.19)] but no association with increasing hours of lifetime pool use (Villanueva et al. 2007). There was no significant difference in risk for ever swimming in pools with any of the genetic polymorphisms under evaluation here. The weak statistical interactions observed with *GSTT1* present (vs. *GSTT1* null) and with *GSTZ1* CT/TT (vs. CC) were in a direction contrary to expectation, given what we observed for interactions with THMs [see Supplemental Material, Table 2A,B (doi:10.1289/ehp.1002206)].

## Discussion

DBPs were previously found to be a bladder cancer risk factor in this case–control study in Spain, as well as in other settings (Villanueva et al. 2004, 2007). In the present study we found significant differences in the dose–response relation of bladder cancer risk with increasing average long-term exposure to DBPs (as represented by THMs) among subjects with differing genotypes in each of three candidate genes. *GSTT1* and *GSTZ1* code for enzymes in the respective biotransformation pathways of two groups of DBPs, the brominated THMs and the α-haloacids. CYP2E1 oxidizes THMs and likely many other compounds in the DBP mixture. Overall, without considering DBP effects or interaction, we found a weak, nonsignificant overall association between polymorphisms in each of these genes and bladder cancer risk. Associations between increasing quartiles of THMs and bladder cancer were stronger among subjects with *GSTT1* +/+ or +/− versus −/−, *GSTZ1* rs1046428 CT or TT versus CC, and *CYP2E1* rs2031920 CC versus CT (no subjects had TT), with statistically significant interactions for each of the respective gene variants. Among the 195 cases and 192 controls with *GSTT1* present and *GSTZ1* rs1046428 CT/TT, ORs (95% CIs) for quartiles 2, 3, and 4 of long-term average THMs were 1.5 (0.7–3.5), 3.4 (1.4–8.2), and 5.9 (1.8–19.0), respectively, relative to quartile 1. We also found that main effect estimates for bladder cancer in association with these polymorphisms varied by THM level, with significant associations for all three high-risk genotypes among subjects within the highest quartile of long-term THMs (Table 4). The supermultiplicative interactions between residential water THM level and polymorphisms in *GSTT1*, *GSTZ1*, and *CYP2E1* are consistent with the hypothesis that these genes influence the metabolism of carcinogens in the DBP mixture in drinking water.

GSTZ1, conserved over a long evolutionary period, plays a key role in the catabolism of phenylalanine and tyrosine. In addition, GSTZ1 transforms several xenobiotic α-haloacid substrates. The HAAs dichloroacetic acid, bromochloroacetic acid, and dibromoacetic acid are transformed to glyoxylic acid, and 2,2-dichloropropanoic acid is metabolized to pyruvic acid (Board and Anders 2005). In rodents, dichloroacetic acid causes liver cancer and dibromoacetic acid is a multisite carcinogen (DeAngelo et al. 1999; Melnick et al. 2007). Among GSTZ-depleted rats, total body clearance of dihaloacetic acids was 3–10 times lower than in rats with normal levels of GSTzeta (Saghir and Schultz 2005). Several SNPs in the *GSTZ1* gene are known (Blackburn et al. 2001). Notably, the rs1046428 T allele has been observed to have low enzymatic activity for transformation of dichloroacetic acid compared with the C allele (Blackburn et al. 2001), consistent with our finding of elevated risk for individuals with this genotype. Many other α-haloacids in the DBP mixture that have not been tested for carcinogenicity may also serve as substrates for GSTZ1 and thereby participate in the interaction we observed. Haplotype analysis for *GSTZ1* did not reveal notable elevated or lowered risk of bladder cancer.

A common polymorphic variant of *GSTT1* is the null form of the allele, which is associated with lack of enzymatic activity. About 20% of Caucasians are homozygous null for this gene (Raimondi et al. 2006). In our control population, 22.5% were homozygous null. Brominated THMs are mutagenic and carcinogenic and are among the most prevalent DBPs in chlorinated drinking water. Pegram et al. (1997) and DeMarini et al. (1997) demonstrated that in *Salmonella* transfected with rat *GSTT1-1+*, brominated THMs are activated to mutagens, and they identified the specific mutagenic transitions (GC→AT) involved. Dibromonitromethane, a DBP that has not been tested for carcinogenicity, is also activated to a mutagen by a transgenic strain of *Salmonella* expressing GSTT1-1 (Kundu et al. 2004). Authors of both *in vivo* and *in vitro* studies have suggested a model whereby brominated THMs, after dermal absorption and inhalation, escape first-pass hepatic metabolism and reach target tissues in the urinary tract where the relative proportion of GSTT1 and oxidative enzymes is more favorable for GSTT1-mediated metabolism (Landi et al. 1999; Ross and Pegram 2003, 2004). In the bladder, the brominated THM could be activated to mutagens in a *GSTT1+* person, leading to increased bladder cancer risk. Findings from experimental exposures to humans are also consistent with our observations. Exposure of humans to BDCM showed that 2 of 10 subjects, each of whom were *GSTT1* null and had low CYP2E1 activity, had the lowest total BDCM metabolism (i.e., the highest blood levels of unmetabolized BDCM) and the highest peak levels of mutagenicity in their urine because of unmetabolized urinary BDCM (Leavens et al. 2007). In that experimental study, dermal exposure to BDCM resulted in blood levels 25–130 times higher than those from oral exposure, indicating that this is a major route of exposure to brominated THM. This is consistent with elevated risk for bladder cancer via showering, bathing, and/or swimming (Villanueva et al. 2007). Supporting our observation of a positive interaction of long-term DBPs with *GSTT1+* is a finding of low risk among populations with the *GSTT1* null genotype from an analysis of international data (Kim et al. 2002). Renal cell carcinoma risk is elevated among *GSTT1+* individuals with occupational exposure to pesticides or to solvents such as trichloroethylene, possibly through a similar mechanism (Buzio et al. 2003; Karami et al. 2008).

The phase I metabolic enzyme CYP2E1 oxidizes a wide variety of alkanes, alkenes, and aromatic and halogenated hydrocarbons and activates many of them to carcinogenic compounds (Bolt et al. 2003). Several chemical species in the DBP mixture are potential substrates of CYP2E1. Our data, revealing a significant interaction between the level of long-term DBP exposure and the −1054C>T (sometimes designated as −1053C>T) rs2031920 SNP of *CYP2E1*, suggests the possibility of carcinogenic activation of one or more constituents of the DBP mixture by the common form of this enzyme. However, we make this observation cautiously. Although CYP2E1 levels are partially determined by genetic factors, the enzyme is highly inducible by alcohol and other factors, and its synthesis can be inhibited by food constituents (Oneta et al. 2002; Perocco et al. 2006). We were unable to control for these latter effects. The relatively small number of subjects with the rs2031920 CT/TT genotype (37 cases, 42 controls) contributed to the relative instability of this finding. In addition, our observation of an OR < 1.0 among the most highly exposed subjects with this genotype suggests a cautious interpretation of the *CYP2E1* findings.

It is unlikely that the aromatic amine substrates for NAT2, found in tobacco products and linked with bladder cancer, are present in the DBP mixture (Richardson 2003). However, DBP mixtures are complex, and we felt it worthwhile to evaluate the possibility of an interaction between DBPs and slow/rapid acetylation genotypes of *NAT2* in risk of bladder cancer. Our expectation of little or no interaction was borne out in the data. The mechanism underlying associations between bladder cancer and the *GSTM1*-null genotype is not yet well understood (Garcia-Closas et al. 2005). Our data indicated that the strength of the association of bladder cancer with *GSTM1* null is not affected by exposure to DBPs.

As our primary metric of exposure to DBPs, we used the long-term average THM concentration at the household level (Villanueva et al. 2007). DBPs are a complex mixture of halogenated organics whose composition and concentration vary in time and space. Hundreds of individual chemical species have been identified (Richardson 2003). THMs and HAAs are the chemical groups found at the highest concentrations in most mixtures, typically accounting for 20–30% of bound halogen. Although there is variability among the chemical components of DBP mixtures, the correlation coefficient between THMs and HAAs is usually > 0.7, and THM levels have been used in many epidemiologic studies as a surrogate for the full mixture. Although THMs and haloacetic acids are the most common chemical species within the DBP mixture, they may not be the most toxic/carcinogenic, and one or more of the polymorphisms of interest may be acting in important ways on other compounds with levels that are correlated with THMs.

Elevated levels of THMs and other DBPs are common in chlorinated swimming pools and cause elevated THM blood levels among swimmers (Aggazzotti et al. 1998). We found no significant interaction of the polymorphisms studied here with ever use of swimming pools. However, the number of pool users was small, findings were not statistically stable, and the relative concentrations of various DBPs in pools differ from those in drinking water, precluding interpretation of these findings.

In this hospital-based study, we had a high response rate among both cases and controls, and all diagnoses were histologically confirmed. Controls were matched to cases on age group, sex, and geographic area of residence at diagnosis. Although matching on area of residence may result in overmatching for type of water, possibly biasing relative risks toward the null, most hospital catchment areas were large enough to provide substantial variability in water sources. We examined residential history since 15 years of age, so that persons who may have had the same water source at the time of the study could easily have lived in other places previously. We conducted this analysis on a subset of the full study population, namely, subjects with genotype data and reliable information on DBP exposures for at least 70% of years between 15 years of age and diagnosis (cases) or interview (controls). We included 680 cases (of 1,219 in the full study) and 714 controls (of 1,271), and associations between bladder cancer and cigarette smoking, long-term exposure to THMs, and the main effects of *NAT2* and *GSTM1* (Garcia-Closas et al. 2005; Samanic et al. 2006; Villanueva et al. 2007) were comparable with those estimated for the full study population, suggesting that this subgroup is comparable with the full set of patients recruited to the study.

Bladder cancer risk has been consistently linked with long-term exposure to DBPs, a complex mixture of halogenated compounds in drinking water created when chlorine is used as a disinfectant (Cantor et al. 1998; King and Marrett 1996; McGeehin et al. 1993; Villanueva et al. 2004). Experimental evidence of mutagenicity and carcinogenicity supports these findings (Komulainen 2004; Richardson et al. 2007). Prior to this study, evidence was considered inadequate to firmly support a causal role for DBPs in the etiology of bladder cancer, primarily for two reasons. First, relative risks reported by most epidemiologic studies have been modest, with ORs typically < 2, posing the possibility that observed associations may be due to bias. Second, there have been inconsistencies in sex-specific findings. The evidence of elevated bladder cancer risk from the present study, based on interaction of DBPs with candidate genes known to be in the biotransformation pathways of known carcinogens and mutagens in the mixture, strengthens the case for a causal interpretation. Because > 20% of the controls in our study population were joint carriers of the high-risk genotypes of the three genes we evaluated, our findings may have significant public health implications in relation to preventing cancers following exposure to these water contaminants. Early replication of these findings in another population is warranted.

## Figures and Tables

**Table 1:** ORs (95% CIs) for bladder cancer for quartile levels of average long-term level of household THMs and for selected SNPs.

Measure	*n* (cases/controls)^a^	OR (95% CI)^b^	*p*-Value
Average THM level at home (μg/L, age 15 to index age)			
≤ 8.0	156/175	1.0	
> 8.0–26.0	153/174	1.2 (0.8–1.9)	
> 26.0–49.0	197/169	1.8 (1.1–2.9)	
> 49.0	174/196	1.8 (0.9–3.5)	
*p*_trend_		0.029	

Polymorphism			
*NAT2*			
Fast	243/299	1.00	
Slow	428/403	1.33 (1.06–1.68)	0.01

*GSTM1*			
Present	257/366	1.00	
Null	420/346	1.77 (1.41–2.22)	< 0.001

*GSTT1*			
Null	136/160	1.00	
Present	542/550	1.21 (0.92–1.59)	0.13

*GSTZ1*			
rs1046428, T82M			
CC	405/402	1.00	
CT	213/213	1.06 (0.82–1.35)	0.67
TT	31/29	1.07 (0.61–1.86)	0.82
rs7975, E32K			
GG	314/330	1.00	
AG	275/276	1.06 (0.83–1.34)	0.66
AA	68/63	1.22 (0.82–1.81)	0.33
rs7972, G42R			
GG	553/572	1.00	
AG	100/91	1.16 (0.84–1.61)	0.36
AA	4/6	0.69 (0.18–2.56)	0.57

*CYP2E1*			
rs2070676			
CC	479/474	1.00	
CG	161/166	0.93 (0.71–1.21)	0.59
GG	9/5	2.03 (0.62–6.62)	0.24
rs2031920			
CC	590/569	1.00	
CT	37/42	0.76 (0.47–1.23)	0.27
TT	0/0	—	
rs8192766			
GG	528/533	1.0	
GT	118/106	1.05 (0.77–1.42)	0.77
TT	3/7	0.32 (0.08–1.30)	0.11

a Numbers of cases or controls per measure may not equal the total number of cases (680) and controls (714) because of missing genotype data.

b OR (95% CI) from logistic regression adjusted for age (continuous), sex, smoking status (never/former/current), size of the municipality of longest residence until 18 years of age, education (three strata), geographic area (six strata), and overall quality of interview.

**Table 2:** Interaction between average THM exposure and polymorphic forms of three genes: *GSTT1*, *GSTZ1*, and *CYP2E1*.^a^

Gene, average THM (μg/L)	*n* (cases/controls)	OR (95% CI)	*n* (cases/controls)	OR (95% CI)	*p*_interaction_^b^
*GSTT1*	Null		Present		

≤ 8.0	34/34	1.0 (reference)	121/141	1.0 (reference)	
> 8.0–26.0	36/37	1.2 (0.5–2.5)	116/136	1.2 (0.7–1.9)	
> 26.0–49.0	37/41	1.2 (0.5–2.5)	160/126	2.0 (1.2–3.4)	
> 49.0	29/48	1.0 (0.4–2.5)	145/147	2.2 (1.1–4.3)	0.021
* p*_trend_		0.28		0.0072	

*GSTZ1* rs1046428	CC		CT/TT		

≤ 8.0	95/86	1.0 (reference)	52/62	1.0 (reference)	
> 8.0–26.0	100/102	1.1 (0.7–1.9)	47/54	1.4 (0.7–2.7)	
> 26.0–49.0	116/97	1.5 (0.9–2.7)	73/62	2.2 (1.1–4.2)	
> 49.0	94/117	1.3 (0.6–2.8)	72/64	2.9 (1.3–6.7)	0.018
* p*_trend_		0.28		0.0043	

*CYP2E1* rs2031920	CT/TT		CC		

≤ 8.0	15/9	1.0 (reference)	125/132	1.0 (reference)	
> 8.0–26.0	10/14	0.98 (0.4–2.5)	133/141	1.3 (0.8–2.0)	
> 26.0–49.0	9/11	1.1 (0.4–3.1)	176/134	2.1 (1.2–3.5)	
> 49.0	3/8	0.6 (0.1–2.7)	156/162	2.0 (1.0–4.1)	0.035
* p*_trend_		0.33		0.014	

a ORs (95% CIs) from logistic regression adjusted for age (continuous), sex, smoking status (never/former/current), size of the municipality of longest residence until 18 years of age, education (three strata), geographic area (six strata), and overall quality of interview.

b *p*-Value for multiplicative interaction between THM level and the respective polymorphism.

**Table 3:** ORs (95% CIs) for the combined effects of polymorphic forms of *GSTT1* and *GSTZ1*.^a^

	*GSTT1* null and *GSTZ1* CC		*GSTT1* present and *GSTZ1* CT/TT		
Average THM	*n* (cases/controls)	OR (95% CI)	*n* (cases/controls)	OR (95% CI)	*p*_interaction_^b^
≤ 8.0	19/17	1.0 (reference)	40/51	1.0 (reference)	
> 8.0–26.0	25/24	1.1 (0.4–3.0)	35/45	1.5 (0.7–3.5)	
26.0–49.0	21/29	1.1 (0.4–3.1)	61/49	3.4 (1.4–8.2)	
> 49.0	17/26	1.5 (0.4–5.4)	59/47	5.9 (1.8–19.0)	
*p*_trend_		0.57		0.0012	0.0052

a OR (95% CI) from logistic regression adjusted for age (continuous), sex, smoking status (never/former/current), size of the municipality of longest residence until 18 years of age, education (three strata), geographic area (six strata), and overall quality of interview. Analysis was restricted to subjects with either both *GSTT1* null and *GSTZ1* rs1046428 CC or both *GSTT1* present and *GSTZ1* rs1046428 CT/TT.

b *p*-Value for multiplicative interaction between the average THM level (age 15 to index year) and the indicated genetic characteristic; the calculation uses the median level of average THM within each successive quartile.

**Table 4:** ORs and 95% CIs for five polymorphic genes (*GSTT1*, *GSTZ1*, *CYP2E1*, *NAT2*, *GSTM1*) within each of four strata of average level of THM in drinking water, in the period from 15 years of age to the index year.^a^

Average THM (μg/L)	*GSTT1* present vs. null	*GSTZ1* rs1046428, CT/TT vs. CC	*CYP2E1* rs2031920, CC vs. CT/TT	*NAT2*, slow vs. rapid/intermediate	*GSTM1*, null vs. present
≤ 8.0	0.9 (0.5–1.5)	0.7 (0.4–1.2)	0.8 (0.5–1.5)	1.3 (0.8–2.0)	1.8 (1.1–2.9)
> 8.0–26.0	0.9 (0.5–1.6)	1.0 (0.6–1.6)	1.2 (0.7–2.0)	1.5 (0.9–2.5)	1.6 (1.0–2.5)
> 26.0–49.0	1.5 (0.9–2.5)	1.0 (0.6–1.6)	1.9 (1.1–3.3)	1.1 (0.7–1.8)	1.8 (1.2–2.8)
> 49.0	1.8 (1.1–3.1)	1.6 (1.0–2.6)	2.0 (1.1–3.9)	1.4 (0.9–2.2)	1.9 (1.2–2.9)

a The referent (OR = 1.0) for each cell in the table, specific to a THM level, is indicated in each column heading. OR (95% CI) from logistic regression adjusted for age (continuous), sex, smoking status (never/former/current), size of the municipality of longest residence until 18 years of age, education (three strata), geographic area (six strata), and overall quality of interview.

## Appendix I Participating Study Centers in Spain

Institut Municipal d’Investigacio Medica, Universitat Pompeu Fabra, Barcelona

Hospital del Mar, Universitat Autonoma de Barcelona, Barcelona

Hospital Germans Trias i Pujol, Badalona, Barcelona

Hospital de Sant Boi, Sant Boi de Llobregat, Barcelona

Consorci Hospitalari Parc Tauli, Sabadell

Centre Hospitalari i Cardiologic, Manresa, Barcelona

Hospital Universitario de Canarias, La Laguna, Tenerife

Hospital Universitario Nuestra Senora de la Candelaria, Tenerife

Hospital General Universitario de Elche, Universidad Miguel Hernandez, Elche, Alicante

Universidad de Oviedo, Oviedo, Asturias

Hospital San Agustin, Aviles, Asturias

Hospital Central Covadonga, Oviedo, Asturias

Hospital Central General, Oviedo, Asturias

Hospital de Cabuenes, Gijon, Asturias

Hospital de Jove, Gijon, Asturias

Hospital de Cruz Roja, Gijon, Asturias

Hospital Alvarez-Buylla, Mieres, Asturias

Hospital Jarrio, Coana, Asturias

Hospital Carmen y Severo Ochoa, Cangas, Asturias
